# Flinders Island Spotted Fever Rickettsioses Caused by “marmionii” Strain of *Rickettsia honei,* Eastern Australia

**DOI:** 10.3201/eid1304.060087

**Published:** 2007-04

**Authors:** Nathan B. Unsworth, John Stenos, Stephen R. Graves, Antony G. Faa, G. Erika Cox, John R. Dyer, Craig S. Boutlis, Amanda M. Lane, Matthew D. Shaw, Jennifer Robson, Michael D. Nissen

**Affiliations:** * Australian Rickettsial Reference Laboratory, Geelong, Victoria, Australia; † Warwick Hospital (Southern Downs Health Services District), Warwick, Queensland, Australia; ‡ Launceston General Hospital, Launceston, Tasmania, Australia; § Fremantle Hospital, Fremantle, Western Australia, Australia; ¶ Menzies School of Health Research, Darwin, Northern Territory, Australia; # University of Queensland, Brisbane, Queensland, Australia; ** Sullivan Nicolaides Pathology, Brisbane, Queensland, Australia; †† Royal Brisbane Hospital, Brisbane, Queensland, Australia

**Keywords:** Spotted fever group rickettsia, *Rickettsia honei*, Flinders Island spotted fever

## Abstract

Australia has 4 rickettsial diseases: murine typhus, Queensland tick typhus, Flinders Island spotted fever, and scrub typhus. We describe 7 cases of a rickettsiosis, with an acute onset and symptoms of fever (100%), headache (71%), arthralgia (43%), myalgia (43%), cough (43%), maculopapular/petechial rash (43%), nausea (29%), pharyngitis (29%), lymphadenopathy (29%), and eschar (29%). Cases were most prevalent in autumn and from eastern Australia, including Queensland, Tasmania, and South Australia. One patient had a history of tick bite (*Haemaphysalis novaeguineae*). An isolate shared 99.2%, 99.8%, 99.8%, 99.9%, and 100% homology with the 17 kDa, *ompA*, *gltA*, 16S rRNA, and Sca4 genes, respectively, of *Rickettsia honei*. This Australian rickettsiosis has similar symptoms to Flinders Island spotted fever, and the strain is genetically related to *R. honei*. It has been designated the “marmionii” strain of *R. honei*, in honor of Australian physician and scientist Barrie Marmion.

## Abstract 2

We report 7 cases of rickettsiosis caused by a new rickettsial strain.

Australia has several endemic rickettsial diseases. In addition, epidemic typhus arrived with the first fleet in 1788 (*1*), but the disease did not become established in Australia. The current endemic rickettsial diseases are murine typhus (*Rickettsia typhi*), scrub typhus (*Orientia tsutsugamushi*), and the spotted fever group (SFG) diseases—Queensland tick typhus (QTT; *R. australis*) and Flinders Island spotted fever (FISF; *R. honei*) (*2*).

QTT, first described in 1946, was characterized as a relatively mild disease with symptoms of fever, headache, malaise, enlarged lymph nodes and a maculopapular (sometimes vesicular) rash. Most patients have an eschar and some have a slight cough, myalgia, and chills (*3*,*4*). Cases of QTT have been detected only on the eastern seaboard of mainland Australia, with most originating in late winter (*5*). FISF was described in Australia, in 1991. It is found in southeastern Australia and is characterized by fever, headache, myalgia, transient arthralgia, maculopapular rash, and cough in some cases (*6*,*7*). Most cases occur in summer. Both QTT and FISF are transmitted to humans by tick bites. Ticks of the genus *Ixodes*, especially *I. holocyclus*, are the main arthropod hosts of QTT and *Bothriocroton hydrosauri* (formerly *Aponomma hydrosauri*) are the main hosts of FISF (*8*–*10*).

We describe 7 cases of a rickettsial disease similar to FISF, which occurred in the eastern half of Australia. The etiologic agent of this disease is an SFG rickettsia, genetically related to *R. honei* and less closely related to *R. australis*. The etiologic agent of the rickettsiosis has been designated the “marmionii” strain of *R. honei*.

## Case Reports

### Patient 1

A 37-year-old woman from Port Willunga, South Australia, sought treatment in February 2003, with a 2-week history of headache, fever, and sweats. No rash or eschar was seen, and she had no recollection of arthropod exposure. She had traveled to Kangaroo Island 2–3 weeks before the onset of illness. Laboratory tests showed elevated levels of liver function test enzymes, mild leukopenia, and thrombocytopenia. Her health improved after receiving oral doxycycline for 5 days. Rickettsial serology later showed an increase in antibody titer. Both PCR and culture results were positive for an SFG rickettsia (Table 1).

**Table 1:** Rickettsial serology, PCR, and culture results from 7 cases of FISF*

		1st serum sample			2nd serum sample			Microbial detection of FISF agent by			
Case	Location	Day after disease onset	SFG titer	TG titer	Day after disease onset	SFG titer	TG titer	Seropositivity	Seroconversion	PCR	Culture
1	Port Wilunga; SA	14	128	<128	190	256	<128	*+*	**–**	*+*	+
2	Darnley Is; QLD	8	256	<128	186	512	<128	*+*	–	*+*	*+*
3	Darnley Is; QLD	3	<128	<128	179	<128	<128	–	–	*+*	*+*
4	Yam Is; QLD	5	<128	<128	515	<128	<128	–	–	*+*	*+*
5	Innisfail; QLD	7	<128	<128	17	4,096	2,048	*+*	+	*+*	*+*
6	Launceston, TAS	34	256	128	60	256	128	*+*	–	*+†*	–
7	Iron Range; QLD	5	<128	<128	18	1,024	<128	+	+	+‡	ND

*FISF, Flinders Island spotted fever; SFG, spotted fever group; TG, typhus group; SA, Is, island; South Australia; QLD, Queensland, TAS, Tasmania; ND: Not done. †PCR positive at both 34 and 60 d after disease onset. ^‡^Performed on serum and real-time PCR positive only.

### Patient 2

A 9-year-old girl sought treatment at the Darnley Island Health Clinic, Torres Strait, Queensland, in February 2003. She was febrile (38.5°C) and reported headache, nausea, and abdominal pain. She had no eschar or rash. She was initially thought to have a viral illness; however, after 3 days she was still febrile (39.0°C), and the provisional diagnosis was changed to scrub typhus; a regimen of oral doxycycline, 100 mg per day, was begun. She was not seen by medical or nursing staff between day 3 and 8 of the illness, but was afebrile and well by day 8. Her SFG title increased, despite a 6-month delay in obtaining the convalescent-phase serum. Results of culture and PCR of the blood sample taken on day 8 were positive for an SFG rickettsia (Table 1).

### Patient 3

A 27-year-old man sought treatment at the Darnley Island Health Clinic in March 2003. His temperature was 37.4°C, and he reported headache, arthralgia, and cough. He exhibited no eschar or rash. The provisional diagnosis was of viral upper respiratory tract infection. He was seen again on days 3 and 4 with persisting symptoms and a sore throat. On the latter visit his condition was diagnosed as tonsillitis, and treatment with penicillin V was begun. Blood tests for malaria and scrub typhus were initiated. He returned on day 29 with fever (37.6°C), cough, pharyngitis, and arthralgia. Results of serologic investigations for *Plasmodium falciparum* and rickettsia (taken on day 3) were negative. Antibiotics were not given because the illness was thought to be viral. His symptoms resolved within the following 2 weeks. Antirickettsial antimicrobial agents were not given at any stage during the illness. Day 3 serum and follow-up serum specimens obtained 6 months later were both negative for rickettsial antibodies; however, results of PCR and culture on the day 3 blood specimen were positive for SFG rickettsiae (Table 1).

### Patient 4

A 10-year-old boy was brought to the Yam Island Health Clinic, Torres Strait, Queensland, in May 2003, five days into an illness with manifestations of fever (38.1°C), headache, and cough. Diagnostic tests for scrub typhus, malaria and leptospirosis were initiated but he was given no specific antimicrobial therapy. Two days later, he seemed improved, and a provisional diagnosis of viral upper respiratory tract infection was made. However, when he was seen on day 14, some symptoms remained (cough and headache), and treatment with amoxicillin was begun. He was well when examined on day 22. At no stage was he given antirickettsial therapy. His day 5 blood sample was negative for SFG/typhus group (TG) rickettsial antibodies, but results of PCR and culture were positive for a SFG rickettsia. Follow-up serum taken 14 months later was negative for rickettsial antibody (Table 1).

### Patient 5

A 50-year-old man was admitted to Innisfail Hospital, Innisfail, Queensland, in June 2003. He reported a 7-day history of fever and rigors and a 4-day history of maculopapular rash. He also reported myalgia, arthralgia, conjunctivitis, swollen hands, dry cough, and constipation. An eschar was found on the right side of his neck. His temperature was 38.5°C and blood pressure 95/60 mm Hg. Serum chemistry showed elevated levels of total bilirubin (23; normal range 2–20 μmol/L), alkaline phosphatase (276; normal range 30–115 units/L), gamma glutamyl transpeptidase (199; normal range 0–70 units/L), aspartate transaminase (AST) (301; normal range 5–40 units/L), alanine transaminase (ALT) (129; normal range 5–40 units/L), and lactate dehydrogenase (LDH) (701, normal range 100–225 units/L). Further investigation showed proteinuria, moderate thrombocytopenia (59; normal range 150–400×10^9^/L), mild neutrophilia with left shift (7.9; normal range 2.0–7.5×10^9^/L), and lymphopenia (0.7; normal range 1.0–4.0×10^9^/L). Examination of convalescent-phase serum showed seroconversion to SFG rickettsia. Results of rickettsial PCR and culture were positive for a member of the SFG (Table 1). He recovered after treatment with oral doxycycline (100 mg twice per day) for 5 days.

### Patient 6

A 33-year-old man from Lilydale, a small town in northeastern Tasmania, sought treatment from his general practitioner in May 2003 (day 1) after a recent fishing trip. His symptoms included fever (38.3°C) and headache. On day 6 the patient was improving but had developed cervical lymphadenopathy. His illness was thought to be viral in origin so he was not treated with any antibiotics. The patient’s condition improved, and he had a symptom-free period of ≈10 days. Fever developed again 33 days after onset of the earlier illness with the same symptoms including aches and pains. Three days latert, he was admitted to Launceston General Hospital. He appeared markedly ill with a blanching maculopapular rash over his trunk, which had not been evident before, inguinal lymphadenopathy, neutropenia (0.9; normal range 2.0–7.5×10^9^/L) and slightly elevated levels of C-reactive protein (10; normal range 0–8 mg/L). At this time the possibility of rickettsial disease was raised, and appropriate tests were performed.

On day 7 after the second onset of fever, the patient was able to work but still felt ill and had a slight fever (37.6°C). On day 27 after the second onset of fever, more rickettsial tests were performed before he received treatment with a 14-day course of doxycycline. He made a complete recovery without further relapse. He showed a raised rickettsial SFG titer and a positive SFG rickettsial PCR results for both blood samples tested (days 34 and 60) (Table 1).

### Patient 7

A 55-year-old man, an entomologist, at Iron Range, Cape York Peninsula in far north Queensland, removed a tick from the left ventrolateral side of his abdomen in late May 2002. Five days after removing the tick (day 5), an influenzalike illness with myalgia and arthralgia developed. On day 6, a high fever developed, and on the following day, he experienced persisting severe lethargy and severe muscle cramps in major muscle groups of his upper and lower legs. On day 8, an eschar appeared at the site of the tick bite. It was oval in shape and ≈150 mm by 75 mm. A widespread maculopapular/petechial rash also appeared over his body. High fever, severe lethargy, and myalgias continued. On day 9, he visited his doctor in Brisbane where the examination confirmed a widespread maculopapular/petechial rash with generalized lymphadenopathy and myalgias affecting large muscle groups. A large eschar was found on his left lower abdomen. An SFG illness was suspected, and treatment with doxycycline, 100 mg twice per day, was begun His doctor reexamined him on day 20, and his condition had improved. His myalgia had decreased, and the rash faded over 5 weeks.

Laboratory testing on day 10 showed lymphopenia (0.8; normal range 1.0–4.0×10^9^/L) and mild thrombocytopenia (146; normal range 150–400×10^9^/L). Liver function tests showed slightly elevated AST (48; normal range 5–40 units/L) and ALT (44, normal range 5–40 units/L) and mildly elevated LDH (325; normal range 100–225 units/L). Rickettsial serology was negative on day 10 but convalescent-phase serology on day 23 showed an SFG seroconversion. A real-time PCR on the day 10 serum specimen showed a positive result for the SFG/TG *gltA* gene, but the 17-kDa PCR result was negative (Table 1).

The removed tick was subsequently identified as *Haemaphysalis novaeguineae.* DNA was extracted from the tick and PCRs performed targeting the rickettsial *rrs*, *ompA* and *ompB* genes. PCR products were sequenced, aligned, searched with BLAST (available from http://130.14.29.110/BLAST/)**,** and submitted to GenBank (accession nos. AJ585043, AJ585044, and AJ585045 for the *rrs*, *ompA,* and *ompB* genes, respectively). Phylogenetic analysis of all 3 genes showed that the closest relatives were *R. honei* strain TT-118 (Thai tick typhus) and *R. honei* strain RB (FISF) (*11*).

## Methods

### Rickettsial Serology

Serologic testing was performed on human serum specimens by using a goat anti-human IgM, IgG, and IgA fluorescein isothiocyanate–labeled secondary antibody (Kirkegaard and Perry Laboratories, Gaithersburg, MD, USA), by an indirect immunofluorescence assay (IFA) as described (*7*). Antigens used included *R. honei*, *R. australis*, *R. akari*, *R. conorii*, *R. sibirica,* and *R. rickettsii* from the SFG; and *R. typhi* and *R. prowazekii* from the TG. All titers >128 were considered positive.

### Rickettsia Isolation from Blood

Rickettsial isolation was performed with Vero cell cultures as previously described (*12*). Cultures were observed microscopically weekly for a cytopathic effect and monthly by immunofluorescence. IFA-positive cultures had their DNA extracted and their rickettsial status confirmed by PCR. Positive cultures were passaged onto confluent XTC-2 cell monolayers and grown at 28°C in Leibovitz L-15 media (Invitrogen, Melbourne, Victoria, Australia) supplemented with 5% heat-inactivated fetal bovine serum, 0.4% tryptose phosphate (Oxoid, Basingstoke, UK), and 200 mmol/L-glutamine (Invitrogen).

### Rickettsial PCR on Blood

Rickettsial real-time PCR was performed on buffy coat (except for serum for case 7). DNA was extracted by using a DNA extraction kit (Gentra, Minneapolis, MN, USA) and the primers CS-F and CS-R and the probe CS-P (Table 2; Biosearch Technologies Inc., Novato, CA, USA) as previously described (*13*).

**Table 2:** Oligonucleotide primers used for PCR amplification and sequencing

Primer	Nucleotide sequence (5′→3′)	Gene	Reference
CS-F	TCG CAA ATG TTC ACG GTA CTT T	*gltA*	*29*
CS-R	TCG TGC ATT TCT TTC CAT TGT G	*gltA*	*29*
CS-P*	TGC AAT AGC AAG AAC CGT AGG CTG GAT G	*gltA*	*29*
MTO-1	GCT CTT GCA ACT CTA TGT T	*orf17*	*12*
MTO-2	CAT TGT TCG TCA GGT TGG CG	*orf17*	*12*
CS-162-F	GCA AGT ATC GGT GAG GAT GTA ATC	*gltA*	*15*
CS-398-SF	5′ATT ATG CTT GCG GCT GTC GG	*gltA*	*15*
CS-731-SR	AAG CAA AAG GGT TAG CTC C	*gltA*	*15*
RpCS1258	ATT GCA AAA AGT ACA GTG AAC A	*gltA*	*17*
rRNA1	AGA GTT TGA TCC TGG CTC AG	*rrs*	*16*
rRNA2	AAG GAG GTG ATC CAG CCG CA	*rrs*	*16*
rRNA3	CCC TCA ATT CCT TTG AGT TT	*rrs*	*16*
rRNA4	CAG CAG CCG CGG TAA TAC	*rrs*	*16*
Rr190.70p	ATG GCG AAT ATT TCT CCA AAA	*ompA*	*17*
Rr190.602n	AGT GCA GCA TTC GCT CCC CCT	*ompA*	*17*
D1f	ATG AGT AAA GAC GGT AAC CT	*sca4*	*18*
D928r	AAG CTA TTG CGT CAT CTC CG	*sca4*	*18*
D767f	CGA TGG TAG CAT TAA AAG CT	*sca4*	*18*
D1390r	CTT GCT TTT CAG CAA TAT CAC	*sca4*	*18*
D1219f	CCA AAT CTT CTT AAT ACA GC	*sca4*	*18*
D1876r	TAG TTT GTT CTG CCA TAA TC	*sca4*	*18*
D1738f	GTA TCT GAA TTA AGC AAT GCG	*sca4*	*18*
D2482r	CTA TAA CAG GAT TAA CAG CG	*sca4*	*18*
D2338f	GAT GCA GCG AGT GAG GCA GC	*sca4*	*18*
D3069r	TCA GCG TTG TGG AGG GGA AG	*sca4*	*18*

*5′ end labeled with 6-FAM; 3′ end labeled with BHQ-1.

Confirmatory PCR was performed on the 17-kDa gene (*orf17*) by using the primers MTO-1 and MTO-2 (Table 2; Invitrogen) (*14*), with an annealing temperature of 51°C and a total of 45 cycles. PCR products were visualized by electrophoreses on a 1% Tris-acetate EDTA agarose gel (Amresco, Solon, CA, USA) stained with ethidium bromide. PCR-positive samples had their DNA cleansed using the QIAquick DNA clean up kit (QIAGEN, Düsseldorf, Germany) and were sequenced at Newcastle DNA (Newcastle University, Newcastle, New South Wales, Australia). Phylogenetic analysis of DNA sequences was performed with DNADIST and NEIGHBOR computer programs of the PHYLIP version 3.63 software package (available from http://evolution.genetics.washington.edu/phylip.html). Sequences were compared to those of the rickettsial strains considered to be valid species (*15*). Phylogenetic trees and bootstrap analyses were performed with 100 alignments by using the SEQBOOT and CONSENSE programs of PHYLIP.

### Rickettsial Molecular Characterization

Rickettsial isolates had portions of their *gltA*, 16S rRNA, ompA, and Sca4 antigen genes amplified and sequenced to supplement the 17-kDa gene analysis done on buffy coat and cultures. The primer pairs CS-162-F with CS-731-SR and CS-398-SF with RpCS1258 (Table 2) were used to amplify the 5′ and 3′ ends of *gltA,* respectively (*16*).

The 16S rRNA gene (*rrs*) was amplified by using the primer pairs rRNA1 with rRNA3 and rRNA2 with rRNA4 (Table 2) (*17*). The PCR contained 1 μmol of each respective primer, 200 μmol/L of each dNTP, 10× reaction buffer, 2 mmol/L MgCl_2_, 2 U Taq polymerase, and 4 μL of rickettsial DNA extract. The amplification was performed in a thermocycler (Rotor-Gene 3000, Corbett Research, Sydney, New South Wales, Australia) with an initial denaturation of 95°C for 3min, followed by 40 cycles of denaturation at 95°C for 30 s, annealing at 51°C for 30 s, and extension at 72°C for 1 min; with a final extension of 10 min. PCR products were visualized and sequenced as described above.

The ompA gene (*ompA*) was amplified by using the primers Rr190.70p and Rr190.602n (Table 2) by using the above protocol but with an annealing temperature of 48°C (*18*). The Sca4 antigen gene (*sca4*) was amplified by using the primer pairs D1f and D928r, D767f and D1390r, D1219f and D1876r, and D1738f and D2482r, following the specified protocol (*19*). The final segment of the gene was amplified with the primers D2338f and D3069r following the same protocol and an annealing temperature of 48°C (*19*).

## Results

Seroconversion, defined as a 4-fold increase in antibody titer, occurred in only 2 of the 7 patients (patients 5 and 7), although positive titers were seen in 5 of 7 patients (Table 1). In 5 of 6 patients a rickettsia was isolated from blood (in EDTA-vacutainers; Table 1) in Vero cell culture, however, 4 of these 5 isolates did not persist in cell culture after their third passage. The remaining isolate, from patient 5, has been maintained in continuous culture in only the XTC-2 cell line.

Patients 1–6 had rickettsial DNA detected in their buffy coat DNA extracts by real-time PCR. Patient 7 had rickettsial DNA detected in his serum by using real-time PCR. Of the 7 cases, all but 1 (patient 7), were PCR positive for the 17-kDa gene and all 5 positive rickettsial cultures were also PCR positive for the same gene (Table 1). The 17-kDa PCR sequences for the buffy coats of cases 1–6 and cultures of patients 1–5 were found to be 100% homologous to one another and to the Japanese *Haemaphysalis* tick sequences Hf151 and Hl550 (*20*) (GenBank accession nos. AB114816 and AB114807, respectively). A 399-bp sequence also exhibited 99.2% homology with *R. honei* strains RB and TT-118 (GenBank accession nos. AF060704 and AF060706, respectively) as shown in the phylogenetic tree (Figure).

**Figure:**
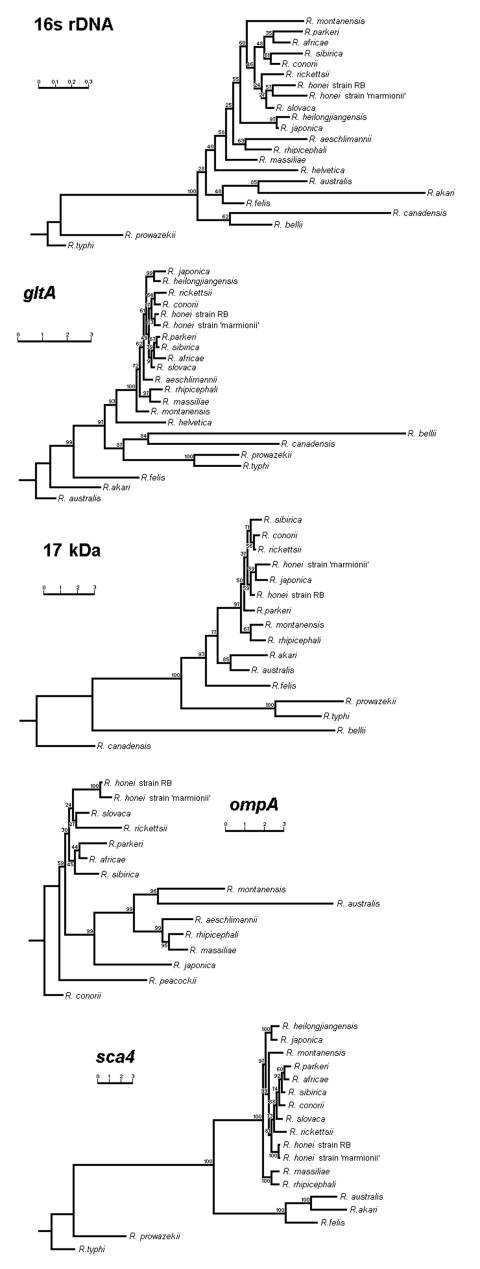
Phylogenetic trees obtained by a neighbor-joining analysis of the 16s RNA, *gltA*, 17-kDa, ompA, and Sca4 antigen genes. Bootstrap values from 100 analyses are shown at the node of each branch.

Analysis of a 1082 bp *gltA* sequence from the KB strain exhibited 99.7 and 99.8% homology with *R. honei* strains RB and TT-118, respectively (GenBank accession nos. AF018074 and U59726, respectively) (Figure). An 1142 bp *rrs* sequence exhibited 100% homology with the Australian *Haemaphysalis novaeguineae* tick sequence AL2003 (*11*) (GenBank accession no. AJ585043) and a 1,388-bp sequence exhibited 99.6% and 99.9% homology with the *R. honei* strains RB and TT-118, respectively (GenBank accession nos. U17645 and L36220, respectively) (Figure). A 511-bp sequence of *ompA* exhibited 100% homology with the *H. novaeguineae* sequence AL2003 (*11*) (GenBank accession no. AJ585044) and a 513-bp sequence had 99.8% homology with the *R. honei* strains RB and TT-118 (GenBank accession nos. AF018075 and U43809, respectively). Only 1 nucleotide substitution only was found in a 2,961-bp sequence of the Sca4 gene (100% homology) with *R. honei* strain RB (GenBank accession no. AF163004) (Figure).

These *R. honei* strain “marmionii” sequences have been submitted to GenBank with the accession nos. AY37683 for the 17-kDa gene, AY37684 for the *gltA* gene, AY37685 for the 16S rRNA gene, DQ309095 for the Sca4 gene, and DQ309096 for the *ompA* gene.

## Discussion

These 7 cases of FISF are 1 of many newly emerging rickettsial diseases (*21*). Its symptoms are consistent with a relatively mild rickettsial SFG disease. The most frequent acute symptoms observed were fever (100%), headache (71%), arthralgia (43%), myalgia (43%), cough (43%), rash (maculopapular/petechial) (43%), nausea (29%), pharyngitis (29%), and lymphadenopathy (29%). In only 2 patients was an eschar evident. The rash did not appear on the palms or soles, unlike previously reported FISF cases (*6*,*12*). One patient (patient 7), had a history of a *H. novaeguineae* tick bite, which may imply an incubation period of 5 days. The cases in this report occurred between February and June (late summer and autumn), in contrast to previously described cases of FISF and QTT, which have their peak onsets in summer and late winter, respectively (*5*,*6*).

The biphasic illnesses seen in patients 3 and 6 were unusual for SFG rickettsial diseases. Because no specimens were taken during the initial phase of either patient’s illness, that this phase was rickettsial in nature cannot be confirmed. Patient 6’s illness may have been rickettsial in nature because of the appropriate incubation time after a fishing trip in an area endemic for ticks. His illness had the longest duration of all the reported cases, with rickettsiae still detectable 27 days after the onset of the second febrile illness. This is possibly the first report of an SFG rickettsia being associated with a chronic infection in a human. Relapsing rickettsial diseases are known to exist, such as Brill disease, a recurrent form of epidemic typhus (*22*). Rickettsiae persisting in human and animal organs after illness have been reported with scrub typhus and SFG rickettsia (*23*,*24*). An Australian case of recurrent rickettsial illness was diagnosed serologically as QTT (*25*).

The isolation of rickettsiae from patient 2 after antimicrobial drug therapy and while she was clinically well is unusual. The presence of rickettsiae may be due to the bacteriostatic nature of the patient’s treatment, which allowed a small number of rickettsiae to survive before being eliminated by her immune system. This phenomenon may also have been the beginning of a chronic infection, as described above in patients 3 and 6.

Apart from patients 5 and 7, antibody levels of paired serum specimens (Table 1) did not show a marked rise in titer. Because the second serum sample from 4 of the case-patients was received in excess of 6 months after illness, the antibody levels may have subsided, explaining the apparent lack of seroconversion in patients 3 and 4. Because most rickettsioses are diagnosed through serologic tests, some cases of rickettsial disease are likely being missed due to a lack of seroconversion, as we have observed with these cases of FISF. This demonstrates the usefulness of PCR for diagnosing acute rickettsial diseases. Cases of rickettsioses without seroconversion or positive serology titers have been previously described with “*R. sibirica mongolotimonae*” (*26*). Despite the initial isolation of *R. honei* strain “marmionii” in Vero and L929 cells at 35°C, no isolate could be continuously grown in these cell lines. This may be due partially to temperature-dependent growth kinetics, similar to those of *R. felis* (*27*).

The 7 described cases were distributed widely throughout eastern Australia. Cases have appeared on the eastern seaboard of Australia (including the Torres Strait), Tasmania, and in South Australia. Cases are yet to be reported in Victoria, New South Wales, the Northern Territory, or Western Australia. The discovery of FISF cases in the Torres Strait suggests its possible presence in Papua New Guinea. In comparison, QTT is found only down the eastern seaboard and not south or west of Wilson’s Promontory in Victoria. Traditionally, FISF has only been found in the southeastern states, including Tasmania and South Australia (*12*,*28*).

At present, *R. honei* has been found on 2 other continents, with potential reservoirs in *Ixodes* and *Rhipicephalus* ticks in Asia and in *Amblyomma cajennense* in North America (*29*). The only known vector/reservoir of *R. honei* in Australia is *Bothriocroton hydrosauri* (*10*). *R. honei* strain “marmionii” has not been found in any *B. hydrosauri* ticks, although *H. novaeguineae* may be a vector/reservoir, as a *H. novaeguineae* tick was removed from patient 7 before the onset of illness. Rickettsial *rrs* and *ompA* gene sequences within the tick demonstrated 100% homology with *R. honei* strain “marmionii” (*11*). *H. novaeguineae* is known to bite numerous animals including humans and is found in both northern Australia and Papua New Guinea (*30*). The vectors and reservoirs of *R. honei* strain “marmionii” in southern Australia are not known.

When compared phylogenetically to other rickettsiae, *R. honei* strain “marmionii” has the closest homology with Australian *R. honei* strain RB, which had been isolated from a febrile patient on Flinders Island. When the *gltA*, *rrs*, *ompA*, *orf17*, and *sca4* genes are compared between *R. honei* strains RB and “mamionii,” they are 99.7%, 99.6%, 99.6%, 99.0%, and 100% homologous, respectively. Homologies of 99.8% and 99.9% are seen with the *gltA* and *rrs* genes, respectively, when *R. honei* strains TT-118 and “marmionii” are compared. An 811-bp *ompB* gene sequence from the *H. novaeguineae* tick removed from patient 7 also showed 100% homology with *R. honei* (*11*). This supports its description as an SFG rickettsia but not a new species by using previously proposed criteria (*15*). Further analysis is needed to further define the taxonomic position of *R. honei* strain “marmionii.”

The 7 cases of an illness similar to FISF demonstrate that new emerging rickettsioses are present in Australia. These described cases encompass a geographic distribution larger than those of FISF and QTT. The only known tick host of *R. honei* strain “marmionii” is *H. novaeguineae*, a tick not previously recognized as a transmitter of human pathogens. Genetically, the etiologic agent of these 7 cases is closely related to *R. honei*. We propose to name the agent *Rickettsia honei* strain “marmionii,” in honor of the Australian physician and scientist Barrie P. Marmion, for his research into Q fever, another important rickettsial disease.
